# Estudo Randomizado Comparando a Ablação por Cateter com o PVAC Gold vs. Tratamento com Fármacos Antiarrítmicos em Pacientes Idosos com Fibrilação Atrial Sintomática

**DOI:** 10.36660/abc.20230684

**Published:** 2024-07-11

**Authors:** Luiz Claudio Berhmann Martins, F Cristiano, Fabio K. Dorfman, Francisco C. C. Darrieux, Tan C. Wu, P Alberto, Denise T. Hachul, Claudio Campi de Castro, Rogério Ruscitto do Prado, Luciana V. F. Souza, Luciana Sacilloto, Gabrielle D. A. Pessente, Cesar J. Grupi, Muhieddine Omar Chokr, Cesar H. Nomura, Kátia Rodrigues de Oliveira, Conrado P. Balbo, Sissy L. Melo, Pedro Veronese, Mauricio I. Scanavacca

**Affiliations:** 1 Hospital das Clínicas Faculdade de Medicina Universidade de São Paulo São Paulo SP Brasil Unidade de Arritmia, Instituto do Coração (InCor), Hospital das Clínicas da Faculdade de Medicina da Universidade de São Paulo, São Paulo, SP – Brasil

**Keywords:** Fibrilação Atrial, Ablação por Cateter, Antiarrítmicos, Qualidade de Vida, Acidente Vascular Cerebral

## Abstract

**Fundamento:**

Não existem estudos randomizados comparando a manutenção do ritmo sinusal após ablação por cateter (AC) em relação ao tratamento com fármacos antiarrítmicos (AA) em pacientes idosos portadores fibrilação atrial (FA) paroxística.

**Objetivos:**

Comparar os resultados clínicos do isolamento das veias pulmonares (VPs) com o cateter PVAC Gold de segunda geração com o uso de AA em idosos com FA paroxística sintomática, recorrente, apesar do uso de fármacos AA.

**Métodos:**

Sessenta pacientes com FA paroxística ≥ 65 anos e sem cardiopatias estruturais foram randomizados para duas formas de tratamento: grupo 1: AC e grupo 2: AA. O desfecho primário foi a taxa livre de recorrência de FA após pelo menos um ano de seguimento. Os desfechos secundários foram: progressão para formas persistentes de FA, impacto na qualidade de vida (QVFA) e complicações. O nível de significância adotado na análise estatística foi de 5% (p<0,05).

**Resultados:**

A taxa livre de recorrência de FA foi de 80% (10% com amiodarona) no grupo AC, após 1,3 procedimentos por paciente e de 65% no grupo AA (60% com amiodarona), (p = 0,119) num seguimento médio de 719 dias (Q1: 566; Q3: 730). A taxa livre de FA persistente foi de 83,4% no grupo AC e de 67,7% no grupo AA (p = 0,073). Ambas as estratégias apresentaram melhora no escore de QVFA durante o seguimento (p < 0,001), sem diferença entre os grupos. Embora sem repercussão clínica ou impacto no teste de avaliação intelectual, 25% dos pacientes do grupo PVAC apresentou sinais de embolização cerebral na RNM cerebral.

**Conclusões:**

Ambas as estratégias para manutenção do ritmo sinusal promoveram melhora na qualidade de vida de pacientes idosos com FA sintomática, sem diferença estatística nos desfechos clínicos preconizados. Estudos adicionais usando tecnologias com melhor perfil de segurança são necessários para avaliar os benefícios da AC em pacientes idosos com FA.

## Graphical abstract

**Figura Central:**
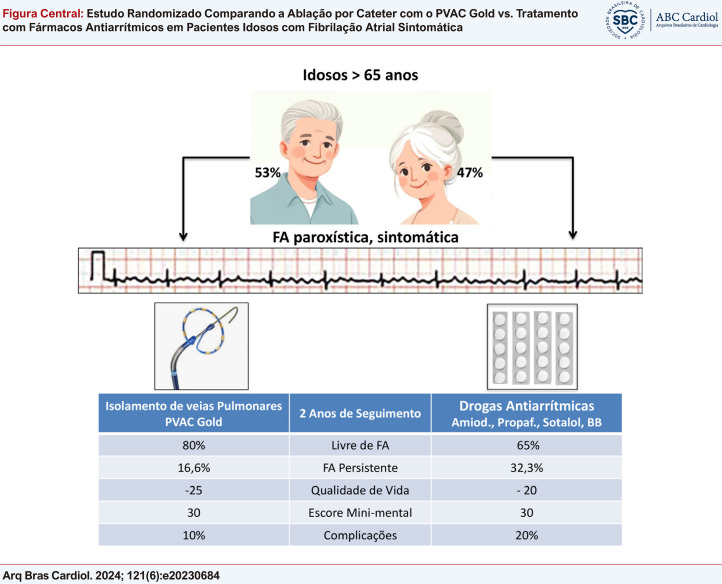
: Estudo Randomizado Comparando a Ablação por Cateter com o PVAC Gold vs. Tratamento com Fármacos Antiarrítmicos em Pacientes Idosos com Fibrilação Atrial Sintomática

## Introdução

O tratamento inicial de idosos com fibrilação atrial (FA) sintomática é usualmente farmacológico, utilizando-se anticoagulantes orais para prevenir acidente vascular cerebral e tromboembolismo sistêmico, associados a fármacos para manutenção do ritmo ou controle da frequência cardíaca.^1,2^

Apesar da ablação por cateter (AC) ser recomendada para pacientes que persistem sintomáticos, por falha ou intolerância ao tratamento clínico,^1,2^ poucas publicações incluíram pacientes idosos, permanecendo sua eficácia e segurança não bem demonstradas em ensaios clínicos randomizados.^3^

O sistema de ablação por radiofrequência PVAC *(“Pulmonary Vein Ablation Catheter”*) Gold foi projetado para realizar o isolamento das veias pulmonares (VPs) de modo rápido e prático e publicações recentes demonstram resultados semelhantes aos outros sistemas em uso clínico.^4,5^

O objetivo deste estudo foi avaliar a efetividade da ablação com o sistema PVAC Gold no controle do ritmo de pacientes idosos com FA sintomática, seu efeito na qualidade de vida e possíveis complicações, tendo como grupo controle, pacientes nas mesmas condições clínicas sob tratamento com fármacos antiarrítmicos (AA).

## Métodos

### População estudada

Pacientes com FA paroxística sintomática, que apresentaram recorrência após o uso de pelo menos um fármaco antiarrítmico, com idade maior ou igual a 65 anos foram incluídos no estudo. Os critérios de exclusão foram: nenhuma tentativa de tratamento clínico anterior ou ablação prévia, FA permanente ou persistente, diâmetro do átrio esquerdo maior que 55 mm, substituição da válvula mitral por prótese mecânica, trombo atrial, outro procedimento intervencionista cardíaco nos últimos 90 dias, embolia cerebral (EC) nos últimos 6 meses, cardiomiopatia estrutural, contraindicações à terapia anticoagulante ou à ressonância magnética cerebral e recusa do paciente em participar do protocolo.

### Delineamento do estudo

O estudo foi prospectivo e randomizado, com o objetivo de comparar o resultado do isolamento das VPs com o cateter PVAC Gold (Medtronic, Inc..) com a terapia farmacológica, em pacientes com FAP sintomática, durante seguimento de pelo menos 1 ano.

Escore de impacto na qualidade de vida (QVFA)^6^ e mini-exame do estado mental (MEEM)^7^ foram aplicados antes do estudo, 6 e 12 meses após inclusão. Um eletrocardiograma (ECG) de 12 derivações foi obtido antes do estudo e em cada visita e o Holter de 24 horas foi realizado depois de 6 e 12 meses.

Os pacientes foram aleatoriamente designados para ablação (grupo AC) ou terapia farmacológica (grupo AA) de acordo com a lista de randomização estratificada e em blocos 1:1 em um sistema computadorizado. Todos os pacientes assinaram termo de consentimento livre e esclarecido em conformidade com a resolução 466/2012 antes da inclusão no estudo. O comitê de ética do centro participante aprovou o estudo, que foi registrado no Clinicaltrials.gov com o identificador NCT04023461.

### Procedimento de ablação

A anticoagulação oral foi requerida por pelo menos 1 mês antes e mantida após a AC. No caso de varfarina, foi considerado adequado o INR entre 2 e 3. Os fármacos AA foram descontinuados por pelo menos 5 meias-vidas (no mínimo uma semana no caso da amiodarona) antes da ablação e mantidos durante 3 meses após a intervenção, período em que as recorrências não foram computadas (*blanking period*).

A ecocardiografia transesofágica foi realizada em todos os pacientes antes da AC para assegurar a ausência de trombo atrial esquerdo. A maioria dos procedimentos de AC foi realizada sob anestesia geral e com monitorização da temperatura esofágica com termômetro único linear, cujo sensor foi posicionado por fluoroscopia em local próximo da área de ablação.

Através de 2 acessos venosos femorais, um cateter decapolar deflectível foi introduzido na veia coronária (Medtronic, Minneapolis, MN, EUA), seguido por uma única punção transeptal usando uma curva fixa de bainha 8.0F (Oscor, Palm Harbor, FL, EUA) guiada por fluoroscopia. No átrio esquerdo, venografia pulmonar foi realizada para definir a localização dos óstios das VPs. A adenosina ou a estimulação ventricular foram utilizadas para reduzir o extravasamento do contraste do átrio esquerdo durante a injeção do contraste iodado.

Heparina na dose de 50-100 UI/kg foi administrada após a punção transeptal e doses adicionais de heparina foram aplicadas durante todo o procedimento para manter o tempo de coagulação ativado em uma faixa terapêutica especificada (>350 s).

O cateter PVAC foi conectado ao gerador de radiofrequência (RF) (software da versão 11 Medtronic Ablation Frontiers GENius MultiChannel RF Generators) GENius (Medtronic, Inc..). A RF foi aplicada por meio da combinação de 1 ou mais dos 5 canais bipolares. Foi realizado monitoramento contínuo da temperatura local cujo alvo era de 60ºC com uma potência máxima de 8 W. A liberação de RF bipolar/unipolar foi ajustada em uma proporção de 4:1 durante os 60 segundos. A interrupção prematura da aplicação foi realizada apenas em caso de dor ou deslocamento do cateter. O isolamento elétrico das VPs, demonstrado pelo bloqueio de entrada e saída, determinou o término do procedimento. Teste de adenosina foi realizado para detectar condução remanescente, e infusão de isoproterenol em bolus de 10, 20 e 30 mcg foram feitas para detectar gatilhos não originados nas VPs. O tempo de procedimento, tempo de ablação, tempo de fluoroscopia, ritmo no início do procedimento, anatomia das VPs, número de aplicações por veia, isolamento por VP, ablações adicionais, cardioversões elétricas e ritmo ao final do procedimento foram anotados. A repetição da ablação foi recomendada se o paciente apresentasse recorrência sintomática de FA, flutter atrial ou taquicardia atrial após o período de *blanking* e o seguimento estendido por mais 12 meses.

### Ressonância magnética cerebral

Ressonância magnética cerebral (1,5-T; Philips Medical Systems, Best, Holanda) foi realizada 24 horas após a ablação. EC foi diagnosticada a partir da detecção de uma anormalidade na sequência de imagens ponderadas por difusão, com um mapa de coeficiente de difusão aparente reduzido. Os infartos cerebrais foram definidos como consequentes a EC quando FLAIR foi positivo, como descrito previamente.^8^

### Endoscopia digestiva alta (EDA)

A EDA foi realizada dentro de 24 horas após procedimento para detectar lesões esofágicas térmicas associadas ao mesmo. Foram categorizadas de acordo com o sistema de Classificação de Kansas City (KCC). Lesões adicionais não relacionadas ao isolamento das VPs também foram descritas.^9^ Na ocorrência de lesões esofágicas, uma endoscopia de controle era realizada uma semana depois, ou até o seu completo desaparecimento. Uma tomografia computadorizada do esôfago foi realizada nos casos com persistência da úlcera após a segunda semana.

### Drogas antiarrítmicas

Amiodarona, sotalol, propafenona, diltiazem e betabloqueadores, isolados ou associados, foram os AA utilizados. As medicações foram trocadas ou suas doses ajustadas durante o período de *blanking*. Caso o paciente não conseguisse manter o ritmo sinusal durante o seguimento, recomendava-se ajuste das doses, substituição das drogas antiarrítmicas (DAA) ou controle da frequência.

### Acompanhamento

Os pacientes foram agendados para retornar à avaliação médica em 1, 3, 6 e 12 meses após a intervenção designada, quando um ECG de 12 derivações foi realizado. O exame físico e os relatos dos pacientes quanto à recorrência de sintomas foram obtidos em cada visita. O escore de QVFA, o Holter de 24 horas e o MEEM foram realizados no início do estudo e aos 6 e 12 meses.

### Desfechos do estudo

O desfecho primário foi a ausência de FA ou taquicardia atrial, com pelo menos 30 segundos de duração, documentada após o período de *blanking* de três meses*.* Desfechos secundários pré-definidos foram: progressão para formas persistentes de FA, impacto na qualidade de vida e complicações. Em caso de recorrência de arritmia atrial documentada por ECG ou Holter, a data do evento foi considerada como o início dos sintomas. Uma complicação maior foi definida como qualquer efeito adverso resultando em morte, lesão permanente ou requerendo tratamento hospitalar.

### Análise estatística

O tamanho da amostra foi calculado para fornecer 80% de poder para mostrar a superioridade da ablação sobre o tratamento medicamentoso em um teste comparando o resultado primário, com um nível alfa de 0,05 bilateral, assumindo probabilidades de recorrência de 65% no grupo de ablação e 30% no grupo de terapia medicamentosa. O tamanho amostral planejado foi de 60 pacientes (30 no grupo AC e 30 no grupo DAA). As características qualitativas foram descritas segundo grupos por meio de frequências absolutas e relativas, e a associação foi verificada com a utilização do teste Qui-quadrado, teste exato de Fisher ou teste da razão de verossimilhança. As variáveis quantitativas foram descritas de acordo com os grupos usando medidas resumo (média ± desvio padrão ou mediana e intervalo interquartil) e comparadas entre os grupos usando o teste t de Student não pareado ou o teste U de Mann-Whitney conforme distribuição de probabilidade avaliado por meio do teste Kolmogorov-Smirnov. Os escores de QVFA foram descritos por grupos ao longo dos momentos de avaliação. As medidas resumo e a comparação entre os grupos e esses momentos foram utilizadas na análise de variância com medidas repetidas, seguida das comparações múltiplas de Bonferroni para verificar entre quais momentos ocorreram as diferenças. As análises de medicamentos específicos no estudo usaram equações de estimativa generalizada com distribuição binomial e função de ligação logit para comparar os grupos durante a avaliação. Foram criadas as curvas de Kaplan-Meier segundo grupos para estimar o tempo de recorrência de fibrilação atrial e comparar os grupos no teste log-rank. Para realizar a análise, foi utilizado o software IBM-SPSS para Windows versão 20.0. Valores de p <0,05 (bicaudal) foram considerados estatisticamente significativos.

## Resultados

Os pacientes foram randomizados entre setembro de 2017 e março de 2020 em um único centro terciário de cardiologia (InCor/Universidade de São Paulo, Brasil). Sessenta pacientes, 30 homens e 30 mulheres, foram incluídos. A Tabela 1 descreve as características da população. Não houve diferenças clínicas significativas entre os grupos, exceto pela maior carga de FA no grupo AC (p <0,001). O tempo médio entre o diagnóstico de FA até o tempo de inclusão foi de 4 anos (Q1: 2; 3:8) em toda a população.

**Tabela 1:** – Características basais dos pacientes

Características	PVAC N = 30	DAA N = 30	p
Idade, anos	71,1 ± 4,0	72,1 ± 5,1	0,40*
Sexo masculino, n (%)	13 (43,3)	15(50)	0,60
Hipertensão, n (%)	27 (90)	30 (100)	0,24
IMC, (Kg/m^2^)	28 ± 4,3	28,1 ± 5,5	0,21*
Diabetes mellitus, n (%)	5 (16,7)	10 (33,3)	0,136
Dislipidemia, n (%)	12 (40)	20 (66,7)	0,038
Anticoagulantes, n (%)	10 (33,3)	15 (50)	0,08&
Varfarina	3 (10)	7 (23,3)	
Dabigatrana	10 (33,3)	6 (20)	
Rivaroxabana	3 (10)	0 (0)	
Edoxabana	4 (13,3)	2 (6,7)	
Prévio AIT/AVC, n (%)	2 (6,7)	3(10)	0,639
Tempo desde diagnóstico de FA, anos (IIQ)	5 (2; 10)	3 (2; 5)	0,12§
Tempo desde último episódio de FA, meses (IIQ)	9,5 (1; 25)	6 (3; 25)	0,76§
Carga de FA nos últimos 12 meses (IIQ)	12 (6; 37,5)	3 (0,5;11)	<0,01§
Drogas antiarrítmicas, n (%)			0,26&
Propafenona	9 (30)	12 (40)	
Somente Betabloqueador	4 (13,3)	4 (13,3)	
Amiodarona	11 (36,7)	13 (43,3)	
Sotalol	4 (13,3)	1 (3,3)	
Cardioversão de FA prévia			0,25&
Química	8 (26,7)	9 (30)	
Elétrica	5 (16,7)	10 (33,3)	
Ambas	1 (3,3)	0 (0)	
Escore de CHA2DS2-VASc, n (%)			0,43&
1	1 (3,3)	0 (0)	
2	9 (30)	9 (30)	
3	14 (46,7)	10 (33,3)	
4	5 (16,7)	8 (26,7)	
5	1 ( 3,3)	3 (10)	
Fração de ejeção do VE, %	64 ± 5	62,8 ± 3	0,28*
Tamanho do AE, mm	41 ± 4	40 ± 6	0,69*

AE: átrio esquerdo; AIT: ataque isquêmico transitório; AVC: acidente vascular cerebral; DAA: FA: fibrilação atrial; IIQ: intervalo interquartil; IMC: índice de massa corporal; PVAC: VE: ventrículo esquerdo. Teste qui-quadrado; & Teste da razão de verossimilhanças; * Teste t-Student não pareado; § Teste Mann-Whitney

### Grupo ablação

Os 30 pacientes randomizados para AC foram submetidos à ablação. A Tabela 2 apresenta as características do procedimento. Em sete (23,1%) pacientes, a ablação do istmo cavo-tricuspídeo foi realizada, além do isolamento das VPs. Nove pacientes (30%) foram encaminhados para repetição do procedimento por recorrência de FA sintomática. Durante a segunda ablação, reconexão das VPs foi observada em todos os pacientes: oito envolvendo as quatro veias e um envolvendo três veias. Nenhum gatilho ectópico fora das VPs foi detectado usando altas doses de isoproterenol.

**Tabela 2:** – Características do procedimento de ablação

Características	N = 15
Tempo de duração de FA, m	155 ± 49
Tempo de FA, m	74 ± 37
Tempo de fluoroscopia, m	13 ±8
Isolamento de todas as 4 VP, n (%)	21 (70)
**Números de aplicações de RF das VP, n (IIQ)**	
VPSE	8.5 (6;10.25)
VPIE	4 (4;7)
VPSD	5 (4;8)
VPID	5 (4;7)
UI de Heparina por pacientes	12. 500 (10 000; 17 125)
Complicações maiores, n (%)	1 (3.3)

*FA: fibrilação atrial; IIQ: intervalo interquartil; UI: unidades internacionais; RF: radiofrequência; VP: veia pulmonar; VPID: veia pulmonar inferior direita; VPIE: veia pulmonar inferior esquerda; VPSD: veia pulmonar superior direita; VPSE: veia pulmonar superior esquerda.*

### Grupo de tratamento com antiarrítmicos

**Figura 1:**
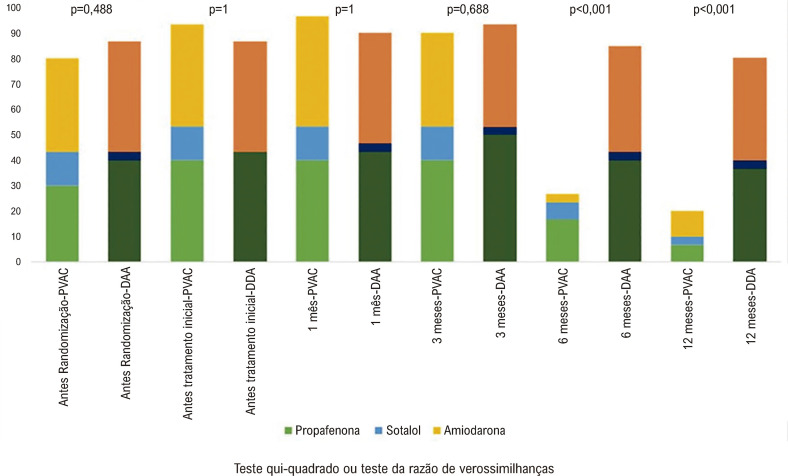
Uso de Antiarrítmicos em Ambos os Grupos em Diferentes Momentos do Seguimento. PVAC: cateter de ablação multipolar de veia pulmonar.

As terapias com DAA ao longo do seguimento e suas respectivas doses estão descritas na Tabela 3 e na Figura 1. Nenhum paciente randomizado para o grupo de DAA foi submetido à AC.

**Tabela 3:** – Uso de antiarrítmicos em ambos os grupos em momentos diferentes

Drogas	PVAC N = 30	AAD N = 30	p
**Propafenona, n (%)**	0,099	0,970	
Antes randomização	9 (30)	12 (40)	0,417
Após tratamento inicial	12 (40)	13 (43,3)	0,793
1 mês	12 (40)	13 (43,3)	0,793
3 meses	12 (40)	15 (50)	0,436
6 meses	5 (16,7)	12 (40)	0,045
12 meses	2 (6,7)	11 (36,7)	0,005
**Sotalol, n (%)**	0,690	0,962	
Antes randomização	4 (13,3)	1 (3,3)	0,148
Após tratamento inicial	4 (13,3)	0 (0)	0,017
1 mês	4 (13,3)	1 (3,3)	0,148
3 meses	4 (13,3)	1 (3,3)	0,148
6 meses	2 (6,7)	1 (3,3)	0,550
12 meses	1 (3,3)	1 (3,3)	1
**Amiodarona,**	<0,001	0,999	
Antes da randomização	11 (36,7)	13 (43,3)	0,598
Após tratamento inicial	12 (40)	13 (43,3)	0,793
1 mês	13 (43,3)	13 (43,3)	1
3 meses	11 (36,7)	12 (40)	0,791
6 meses	1 (3,3)	12 (41,4)	<0,001
12 meses	3 (10)	12 (40)	0,007
**Qualquer AAD, n (%)**	<0,001	0,952	
Antes da randomização	24 (80)	26 (86,7)	0,488
Após tratamento inicial	26 (86,7)	26 (86,7)	1
1 mês	27 (90)	27 (90)	1
3 meses	26 (86,7)	27 (90)	0,688
6 meses	8 (26,7)	25 (83,3)	<0,001
12 meses	6 (20)	24 (80)	<0,001

*Equações de estimativa generalizada com distribuição binomial seguido de comparações múltiplas de Bonferroni. P valor comparação entre os momentos de cada droga. AAD: apêndice atrial direito; PVAC: cateter de ablação multipolar de veia pulmonar.*

### Desfecho primário

**Figura 3:**
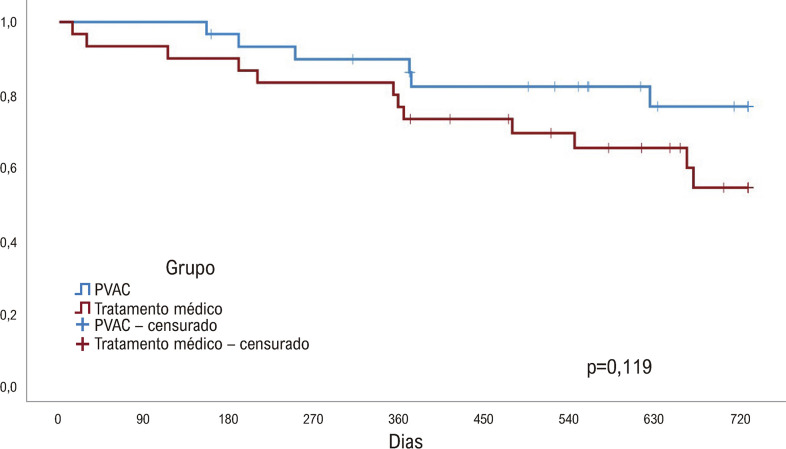
– Curva de Kaplan–Meier da recorrência da fibrilação atrial no acompanhamento de 2 anos, valor p: teste de log-rank. PVAC: cateter de ablação multipolar de veia pulmonar.

No grupo AC, nove (30%) pacientes foram submetidos a um segundo procedimento: oito pacientes foram submetidos à mesma técnica (PVAC Gold) e um à técnica convencional de isolamento de VP com cateter de 8,0 mm dirigido pelo cateter Lasso (J&J). Após uma segunda AC, dois pacientes ainda apresentavam recorrência de FA e foram mantidos com medicação antiarrítmica. Ao final de um acompanhamento médio de 719 dias (Q1: 566; Q3: 730) seis (20%) pacientes no grupo AC e 12 (40%) pacientes no grupo DAA (p = 0,119; Figura 3) apresentaram recorrência de FA. A maioria dos pacientes do grupo AC suspenderam as DAA durante o acompanhamento. Ao final do seguimento de 12 meses, 20% do grupo AC havia recebido DAA (classe IC ou III) em comparação com 80% do grupo DAA (p < 0,001). No grupo AC, houve tendência (p =0,099) à redução no uso de propafenona e redução significativa no uso de amiodarona (p <0,001). As taxas de uso de DAA nos primeiros 12 meses de acompanhamento estão representadas na Tabela 3 e na Figura 1.

### Desfechos secundários

**Figura 2:**
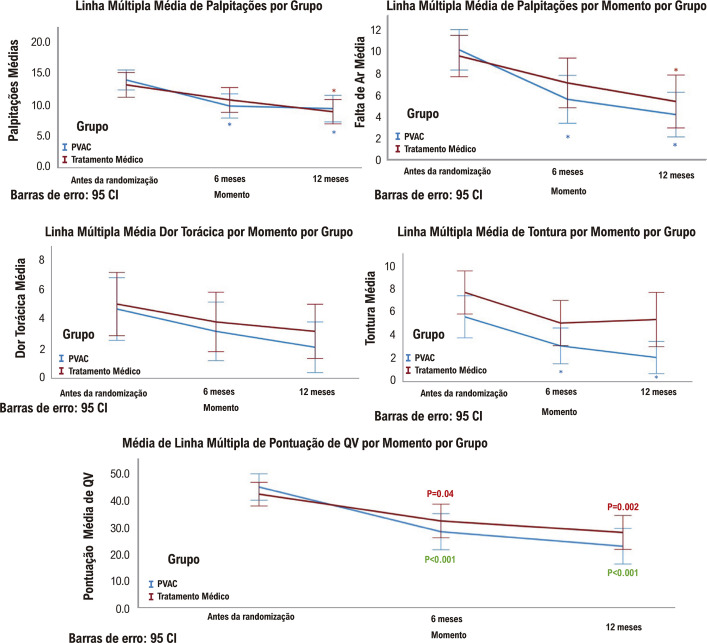
– Valor médio da pontuação do QVFA, * significa valor de p < 0,05. PVAC: cateter de ablação multipolar de veia pulmonar.

Um paciente do grupo AC e 5 pacientes do grupo DAA evoluíram para FA persistente (p =0,073) ao final do seguimento. Melhora significativa na pontuação do QVFA foi observada em ambos os grupos nas avaliações de pontuação de 6 e 12 meses em comparação com suas avaliações basais (Figura 2). Os escores de palpitações e falta de ar melhoraram em ambas as avaliações do grupo PVAC, mas apenas aos 12 meses no grupo de tratamento clínico. Os pacientes sem recorrência de FA apresentaram tendência de melhora no escore de palpitação (p =0,052) e no escore global (p =0,062) no seguimento de 12 meses. Admissão hospitalar para realização de cardioversão elétrica ou farmacológica ocorreu em sete pacientes do grupo AC e três pacientes do grupo DAA (p =0,166). Não houve mudança na pontuação do teste intelectual em relação ao início da randomização em comparação aos acompanhamentos de 6 e 12 meses. (Figura 2)

### Complicações

Não foi possível constatar diferenças estatisticamente significativas entre os dois grupos no critério complicação maior: três pacientes do grupo ablação (10%), em comparação com seis pacientes (20%) do grupo clínico, p =0,472. Nenhum paciente submetido a AC apresentou derrame pericárdico durante o procedimento. Uma paciente foi submetida a cardioversão elétrica devido a taquicardia ventricular sustentada instável relacionada a embolia gasosa durante o acesso transeptal. Um paciente evoluiu com disfunção do nó sinusal e foi tratado com implante de marca-passo definitivo. Não houve complicações vasculares ou lesões do nervo frênico imediatamente após o procedimento. Oito pacientes (26,6%) submetidos à ablação por PVAC Gold apresentaram imagens radiológicas compatíveis com EC sem manifestação de sintomas neurológicos. Uma paciente com embolização gasosa também apresentou imagem de isquemia cerebral na RNM, com reversão completa dos sintomas em cinco dias. Um paciente apresentou perda transitória da visão provocada por oclusão da veia da retina esquerda um dia após isolamento das VPs (excluída embolia arterial). A RNM cerebral foi normal e houve resolução dos sintomas em 30 dias. Três pacientes desenvolveram úlceras esofágicas, KCC 2B (9,9%), mas nenhum paciente desenvolveu fístula atrioesofágica. Entretanto, uma paciente de 68 anos, apresentou úlcera não cicatrizada na segunda EDA, sendo internada e tratada com antibioticoterapia e nutrição parenteral por 7 dias, com resolução completa da lesão

Durante o tempo médio de 12 meses, seis (10%) pacientes do grupo clínico tiveram que interromper ou trocar a DAA devido a efeitos colaterais. Quatro (6,6%) pacientes em uso de amiodarona e um com propafenona associada a betabloqueador. Os principais efeitos colaterais da suspensão do antiarrítmico foram bradicardia (cinco pacientes -16,6%) hipertireoidismo (um paciente - 3,3%).

## Discussão

De nosso conhecimento, este é o primeiro estudo randomizado comparando os resultados da ablação de FA paroxística com o uso de medicações antiarrítmicas em pacientes idosos. O principal achado deste estudo foi que o isolamento das VPs com o cateter PVAC Gold foi eficaz na manutenção do ritmo sinusal na maioria dos pacientes idosos; apenas um paciente evoluiu para FA persistente e houve impacto favorável na qualidade de vida. Entretanto, a ablação com o cateter PVAC Gold não apresentou superioridade estatística quando comparada com o tratamento com medicações antiarrítmicas. Adicionalmente, 30% necessitaram de nova intervenção após os três primeiros meses da ablação, 20% deles ainda com necessidade de introdução de medicação para manutenção do ritmo, três apresentaram complicações com repercussão clínica e 25% apresentaram sinais de embolização cerebral na RNM cerebral.

Os benefícios da manutenção do ritmo sinusal, com ablação ou DAA, não somente para alívio dos sintomas, tem sido debatido há longo tempo, em particular em pacientes mais idosos.^10^O estudo EAST-AFNET 4 mostrou recentemente que a estratégia de iniciar precocemente a terapia para manutenção do ritmo (principalmente com DAA) em pacientes com FA foi associada a um menor risco de morte por causas cardiovasculares, acidente vascular cerebral, hospitalização por insuficiência cardíaca ou síndrome coronariana aguda em relação ao controle da frequência cardíaca, durante um período de acompanhamento de mais de 5 anos.^11^ Consequentemente, as diretrizes de manejo da FA atuais recomendam que a manutenção do ritmo sinusal seja considerada com uso de fármacos AA e/ou AC em pacientes com FA para melhor controle dos sintomas e preservação da condição clínica em longo prazo.^1,2^

Todavia, os efeitos benéficos da ablação de FA na população idosa ainda apresentam aspectos controversos.^3^ Uma análise de subgrupo pré-especificada do estudo CABANA revelou taxas de recorrência de FA menores com ablação do que com terapia medicamentosa nos três subgrupos de idade avaliados, com taxa de risco ajustada (aHR) de 0,47 (IC de 95%, 0,35–0,62) para pacientes com menos de 65 anos, de 0,58 (95% CI, 0,48–0,70) para aqueles entre 65 e 74 anos e de 0,49 (95% CI, 0,34–0,70) para os com mais de 75 anos, sugerindo que ablação de FA seja superior ao tratamento antiarrítmico na prevenção de recorrência independentemente da idade.^12^ Entretanto, as diretrizes atuais sugerem cautela ao considerar a ablação em pacientes idosos devido à carência de dados conclusivos.^1,2^

Em nosso estudo, apesar das curvas comparativas das taxas livre de recorrência de FA nos dois grupos de tratamento se afastarem ao longo do seguimento (Figura 3), não houve diferença estatística entre os pacientes tratados pela ablação (80%) em relação ao tratamento antiarrítmico (65%, p = 0,119). Isso se deveu ao número insuficiente de pacientes envolvidos no estudo, calculados com base nos resultados de estudos randomizados prévios que estudaram pacientes mais jovens e que sugeriam taxa livre de recorrência no grupo ablação de 65% e de 30% nos pacientes sob tratamento clínico.^13^

Apesar das taxas livres de recorrências do grupo ablação apresentarem os resultados esperados, este não foi o caso do grupo clínico, que apresentou resultados superiores aos obtidos pelos controles históricos.^13^ Uma possível explicação para esse achado é que as drogas de classe IA, IC e o cloridrato de sotalol foram os principais AA utilizados nos estudos como base para o cálculo da amostra, e a amiodarona evitada. Por outro lado, em nosso estudo, 40% dos pacientes foram tratados com amiodarona durante o seguimento. Sabe-se que a amiodarona é duas vezes mais eficiente quando comparada à propafenona e ao sotalol, apesar dos efeitos colaterais e de uma taxa considerável de interrupção da droga durante o seguimento de longo prazo.^14^ A adesão medicamentosa também tem sido uma limitação para o tratamento clínico no mundo real. Assim, uma influência adicional para a eficácia do DAA pode estar relacionada ao “efeito do ensaio controlado”. Por fim, os pacientes do grupo DAA apresentaram melhor perfil clínico quanto ao número de recorrências de FA antes da inclusão, apesar da randomização.

Em relação a qualidade de vida avaliada por meio do escore QVFA, o grupo da AC demonstrou uma tendência de melhora no escore de palpitação (p = 0,052) e no escore global (p = 0,062), sendo em sua maioria sem DAA. Este é um achado importante porque a manutenção do ritmo sinusal com DAA pode representar um problema clínico para pacientes idosos, pois além das DAA terem potencial para desenvolverem efeitos adversos e interações medicamentosas, estes pacientes muitas vezes têm que usar vários medicamentos para tratar comorbidades adicionais, apresentam bradicardia sinusal significativa, distúrbios de condução atrioventricular e intraventricular ou disfunção ventricular, que são limitações comuns para uso de DAA nesta população.^15^

EC assintomática espontânea tem sido apontada como causa de demência em pacientes com FA, e declínio neurocognitivo já tendo sido relatada após AC para tratamento de pacientes com FA.^16^ No entanto, mesmo sem sinais e sintomas neurológicos documentados, a consequência clínica dessas lesões ainda é incerta. Contudo, a não manutenção do ritmo sinusal, independente do procedimento de AC, também está relacionada com declínio cognitivo e até evolução para demência vascular.^17^ A EC após-ablação tem sido investigada por diversas técnicas que apresentam grande variação na incidência, de 1,7% até quase 40%.^18^

Um dos motivos de escolhermos o cateter PVAC Gold neste estudo foi o resultado do estudo PRECISION GOLD que utilizou este cateter e demonstrou baixa incidência (2,1%) de EC assintomática, sem mostrar impacto nos testes neurocognitivos.^19^Entretanto, estudo publicado posteriormente ao início do nosso trabalho por Keçe et al.,^20^ documentou incidência de 23% de EC assintomática nos pacientes submetidos ao isolamento das VPs com o cateter PVAC Gold em comparação com 6% nos pacientes submetidos a ablação ponto a ponto com o cateter Thermocool (p = 0,042). Nesse estudo também não houve diferença entre os grupos em relação aos testes neuropsicológicos realizados no seguimento.^20^

A interpretação do risco de EC assintomática fica mais complexa quando analisamos o estudo de Tokuda et al que compararam a incidência de EC em 462 pacientes (média de idade de 59±10 anos) submetidos a AC de FA paroxística com diferentes tecnologias: RF ponto a ponto (193), crioablação por balão (168), RF por balão (“Hotballoon” - 50) e laser por balão (51). A RNM cerebral também foi realizada um dia após o procedimento e mostrou EC assintomática em 30%, 24%, 34% e 39% nos respectivos grupos, com a crioablação apresentando menor taxa de embolização quando comparada com ablação com o balão de laser, mas sem diferença estatística com as técnicas de RF (p = 0.14). Neste estudo também foi realizada análise multivariada, que evidenciou relação entre avanço da idade e aumento da ocorrência de EC (p <0,001).^21^ Esses estudos mostram que existe grande variabilidade na ocorrência de lesões cerebrais após a AC com a utilização da mesma tecnologia, sugerindo que os achados podem depender da técnica de realização da ablação pelo grupo de intervenção. Em nossa série, um paciente (3,3%) apresentou sintomas neurológicos transitórios que desapareceram completamente em 5 dias, mas a imagem cerebral persistiu na ressonância magnética de controle realizada uma semana depois.

Lesões esofágicas silenciosas após AC podem evoluir para fístula atrioesofágica, uma complicação grave, rara e temida. Para evitá-la, várias estratégias têm sido usadas e, embora controversas, o método mais utilizado tem sido o monitoramento da temperatura esofágica e ajustes de potência durante a aplicação de RF na parede posterior do átrio esquerdo.^22^ Estudos recentes com estes ajustes para minimizar lesões esofágicas térmicas mostram que ainda assim ocorrem em torno de 20% nas diversas técnicas de AC, sendo na grande maioria das vezes leves e de resolução espontânea.^23,24^ No presente estudo, detectamos úlceras esofágicas assintomáticas 2B em três (9,9%) pacientes durante endoscopia sistemática realizada após o procedimento. Em um (3,3%) paciente, a úlcera progrediu na avaliação endoscópica da segunda semana, e embora assintomático o paciente foi internado para tratamento hospitalar com cicatrização completa durante o acompanhamento endoscópico.

Nosso estudo também mostrou que três (9,9%) pacientes idosos apresentaram alguma complicação com necessidade de internação hospitalar após AC com PVAC Gold. Essa taxa de complicações parece ser superior quando comparadas à ablação por RF tradicional e à crioablação.^25^Trinta por cento dos pacientes submetidos a AC PVAC Gold precisaram repetir o procedimento de ablação devido a recorrências sintomáticas de FA. Como observado em outros estudos PVAC Gold, a reconexão das VPs esteve presente em todos os pacientes em que o procedimento foi repetido.^26^ Essa é uma questão importante, pois os pacientes idosos não são propensos a tolerar novas internações e procedimentos repetidos. Assim, estratégias mais eficazes para promover isolamento duradouro das VPs já no primeiro procedimento são desejáveis, principalmente nos pacientes idosos.

### Implicações clínicas

Vários estudos randomizados e controlados mostraram que a ablação da FA é segura e superior aos fármacos AA na manutenção do ritmo sinusal. Metanálises recentes demonstraram a superioridade da ablação em relação ao tratamento clínico, inclusive na primeira indicação de tratamento de pacientes com FA.^27,28^ No entanto, vantagem evidente da ablação não tem sido demonstrada em pacientes mais idosos.^3^ A maior incidência de cardiopatias, comorbidades e a baixa taxa de inclusão desses pacientes nos ensaios clínicos randomizados foram consideradas as possíveis razões para esses achados.^3,13^ Assim, investigações mais específicas sobre o papel do ablação nesse importante subgrupo de pacientes são ainda necessárias, principalmente nessa era de introdução de novas tecnologias que inspiram maior segurança mantendo a efetividade.^29^ Nesse sentido, os dados obtidos em nosso estudo podem ser úteis para novas investigações nesta área.

### Limitações

O tamanho amostral do estudo não foi suficiente para demostrar a superioridade da AC, observação comum nos estudos realizados em pacientes mais jovens. A monitorização das recorrências de taquiarritmias com um dispositivo implantado poderia oferecer a carga de FA nas diferentes estratégias, com impacto na progressão da doença e na qualidade de vida dos pacientes. A ausência de um grupo sob controle da FC limita a avaliação do benefício da melhora de qualidade de vida nos dois grupos. O escore MEEM apresenta limitações na avaliação da cognição, principalmente no que diz respeito à função executiva, e pode não ter captado diferenças cognitivas mais sutis. Apesar de estudos prévios apresentarem semelhança de resultados na comparação do PVAC GOLD com ablação com RF ponto a ponto, com benefício de maior rapidez com o PVAC GOLD,^4,5^ durante a realização deste estudo foram publicados outros dois estudos randomizados que demonstraram maior incidência de embolização cerebral assintomática e menor efetividade da ablação com o cateter PVAC Gold em relação a ablação com RF ponto a ponto de nova geração.^20,30^

## Conclusões

A AC com o PVAC Gold e o uso de fármacos AA promoveram manutenção do ritmo sinusal na maioria dos pacientes idosos com FA paroxística sintomática, sem diferença estatística entre si. Ambos os grupos apresentaram melhoria nos escores de qualidade de vida, sem diferença estatística na taxa de progressão para FA persistente ou de complicações. Entretanto, os pacientes submetidos a ablação com PVAC Gold apresentaram taxa de embolização cerebral assintomática maior que a esperada. Estudos adicionais usando tecnologias com melhor perfil de segurança são necessários para avaliar os benefícios da AC em pacientes idosos com FA.
