# Supplementary material for: Estudo Randomizado Comparando a Ablação por Cateter com o PVAC Gold vs. Tratamento com Fármacos Antiarrítmicos em Pacientes Idosos com Fibrilação Atrial Sintomática
Source: Arq Bras Cardiol. 2024 Jul 11;121(6):e20230684. [Article in Portuguese] doi: 10.36660/abc.20230684 (PMC12080719; doi:10.36660/abc.20230684)
Supplement: Translation

# Translation: Randomized Study Comparing Radiofrequency Ablation with the PVAC Gold System vs. Antiarrhythmic Drugs in Elderly Patients with Symptomatic Atrial Fibrillation

**DOI:** 10.36660/abc.20230684i

**Authors:** Luiz Claudio Berhmann Martins, Cristiano F. Pisani, Fabio K. Dorfman, Francisco C. C. Darrieux, Tan C. Wu, Alberto P. Ferraz, Denise T. Hachul, Claudio Campi de Castro, Rogério Ruscitto do Prado, Luciana V. F. Souza, Luciana Sacilloto, Gabrielle D. A. Pessente, Cesar J. Grupi, Muhieddine Omar Chokr, Cesar H. Nomura, Kátia Rodrigues de Oliveira, Conrado P. Balbo, Sissy L. Melo, Pedro Veronese, Mauricio I. Scanavacca

**Affiliations:** 1 Brazil Unidade de Arritmia, Instituto do Coração (InCor), Hospital das Clínicas da Faculdade de Medicina da Universidade de São Paulo, São Paulo, SP – Brazil

**Keywords:** Atrial Fibrillation, Catheter Ablation, Anti-Arrhythmia Agents, Quality of Life, Stroke

## Abstract

**Background:**

There are no randomized studies comparing the maintenance of sinus rhythm after catheter ablation (CA) concerning treatment with antiarrhythmic drugs (AA) in elderly patients with paroxysmal atrial fibrillation (AF).

**Objectives:**

To compare the clinical results of pulmonary vein (PV) isolation with the second-generation PVAC Gold catheter against AA treatment in elderly people with recurrent symptomatic paroxysmal AF, refractory to at least one AA, and without structural heart disease.

**Methods:**

Sixty patients with paroxysmal AF ≥ 65 years old were randomized to two forms of treatment: group 1: CA and group 2: AA drugs. The primary outcome was the AF recurrence-free rate after at least one year of follow-up. Secondary outcomes were: progression to persistent forms of AF, impact on quality of life (QOLF), and complications. The significance level adopted in the statistical analysis was 5% (p<0.05).

**Results:**

The AF recurrence-free rate was 80% (10% with amiodarone) in the CA group, after 1.3 procedures per patient and 65% in the AA group (60% with amiodarone), (p = 0.119) in an average follow-up of 719 days (Q1: 566; Q3: 730). The persistent AF free rate was 83.4% in the AC group and 67.7% in the AA group (p = 0.073) Both strategies showed an improvement in the AFQoL score during follow-up (p < 0.001), with no difference between the groups. Although without clinical repercussions or impact on the intellectual assessment test, 25% of patients in the CA group showed signs of cerebral embolization on brain MRI.

**Conclusions:**

Both strategies for maintaining sinus rhythm promoted an improvement in the quality of life of elderly patients with symptomatic AF, with no statistical difference in the clinical outcomes. Additional studies using technologies with a better safety profile are needed to evaluate the benefits of CA in elderly patients with AF.

## Introduction

The initial treatment of elderly people with symptomatic atrial fibrillation (AF) is usually pharmacological, using oral anticoagulants to prevent stroke and systemic thromboembolism, associated with drugs to maintain the sinus rhythm or control the heart rate.^1,2^

Although catheter ablation (CA) is recommended for patients who remain symptomatic due to failure or intolerance to clinical treatment,^1,2^ few publications have included elderly patients, and its efficacy and safety remain poorly demonstrated in randomized clinical trials.^3^

The PVAC (“Pulmonary Vein Ablation Catheter”) Gold radiofrequency ablation system was designed to isolate PVs quickly and practically and publications demonstrate results like other systems in clinical use.^4,5^

The objective of this study was to evaluate the effectiveness of ablation with the PVAC Gold system in controlling the rhythm of elderly patients with symptomatic AF, its effect on the quality of life, and possible complications, using as a control group patient with the same clinical conditions undergoing treatment with drugs. antiarrhythmics.

## Methods

### Population studied

Patients with symptomatic paroxysmal AF, who had recurrence after the use of at least one antiarrhythmic drugs (AA), aged 65 years or older, were included in the study. Exclusion criteria were no previous clinical treatment attempt or previous ablation, permanent or persistent AF, left atrium diameter greater than 55 mm, mitral valve replacement with a mechanical prosthesis, atrial thrombus, other cardiac interventional procedure in the last 90 days, cerebral embolism (CE) in the last 6 months, structural cardiomyopathy, contraindications to anticoagulant therapy or brain MRI and patient refusal to participate in the protocol.

### Study design

The study was prospective and randomized, intending to compare the results of PV isolation with the PVAC Gold catheter (Medtronic, Inc..) with pharmacological therapy, in patients with symptomatic FAP, during a follow-up period of at least 1 year.

QOLF score^6^ and mini-mental state examination (MMSE)^7^ were applied before the study, 6 and 12 months after inclusion. A 12-lead electrocardiogram (ECG) was obtained before the study and at each visit, and 24-hour Holter monitoring was performed after 6 and 12 months.

Patients were randomly assigned to ablation (Ablation group) or pharmacological therapy (AA group) according to the 1:1 stratified block randomization list in a computerized system. All patients signed an informed consent form following resolution 466/2012 before inclusion in the study. The ethics committee of the participating center approved the study, which was registered on Clinicaltrials.gov with the identifier NCT04023461.

### Ablation procedure

Oral anticoagulation was required for at least 1 month before and continued after CA. In the case of warfarin, an INR between 2 and 3 was considered adequate. AAs were discontinued for at least 5 half-lives (at least one week in the case of amiodarone) before ablation and maintained for 3 months after the intervention, a period in which recurrences were not computed (blanking period). Transesophageal echocardiography was performed in all patients before CA to ensure the absence of left atrial thrombus. Most CA procedures were performed under general anesthesia and with a linear probe thermometer, whose sensor was positioned by fluoroscopy in a location close to the ablation area. Through two femoral venous accesses, a deflectable decapolar catheter was introduced into the coronary vein (Medtronic, Minneapolis, MN, USA), followed by a single transseptal puncture using a fixed curve 8.0F sheath (Oscor, Palm Harbor, FL, USA) guided by fluoroscopy. In the left atrium, pulmonary venography was performed to define the location of the pulmonary vein (PV) ostia. Adenosine or ventricular pacing was used to reduce contrast extravasation from the left atrium during iodinated contrast injection. A bolus of heparin (50-100 IU/kg) was administered after a transseptal puncture, and additional doses of heparin were applied throughout the procedure to maintain the activated clotting time in a specified therapeutic range (>350 s). The PVAC catheter was connected to the RF generator (Medtronic Ablation Frontiers GENius MultiChannel RF Generators version 11 software) GENius (Medtronic, Inc..). RF was applied by combining 1 or more of the 5 bipolar channels. Continuous local temperature monitoring was carried out with a target of 60ºC with a maximum power of 8 W. The bipolar/unipolar RF release was adjusted in a 4:1 ratio during the 60 seconds. Premature interruption of the application was conducted only in case of pain or displacement of the catheter. The electrical isolation of the PV, demonstrated by the entry and exit blockage, determined the end of the procedure. An adenosine test was performed to detect remaining conduction, and isoproterenol infusion bolus of 10, 20, and 30 mcg were performed to detect non-PV triggers. The procedure time, ablation time, fluoroscopy time, rhythm at the beginning of the procedure, PV anatomy, number of applications per vein, PV isolation, additional ablations, electrical cardioversions, and rhythm at the end of the procedure were noted. Repeat ablation was recommended if the patient experienced a symptomatic recurrence of AF, atrial flutter, or atrial tachycardia after the blank period and up to a period of 6 months. In those cases, the follow-up was extended for another 12 months.

### Brain MRI

Brain MRI (1.5-T; Philips Medical Systems, Best, Netherlands) was performed 24 hours after ablation. CE was diagnosed based on the detection of an abnormality in the diffusion-weighted imaging sequence, with a reduced apparent diffusion coefficient map. Cerebral infarcts were defined as resulting from CE when FLAIR was positive, as previously described.^8^

### Upper Digestive Endoscopy (EDA)

EDA was performed within 24 hours after the procedure to detect thermal esophageal injuries associated with the procedure. They were categorized according to the Kansas City Classification (KCC) system. Additional lesions unrelated to PV isolation were also described.^9^ In the event of esophageal lesions, a control endoscopy was performed one week later, or until their complete disappearance a computed tomography of the esophagus was performed in cases with persistence of the ulcer after the second week.

### Antiarrhythmic drugs

Amiodarone, sotalol, propafenone, diltiazem, and beta-blockers, alone or in combination, were the antiarrhythmic drugs used. Medications were changed or doses adjusted during the blanking period. If the patient was unable to maintain sinus rhythm during follow-up, dose adjustments, AA replacement, or frequency control were recommended.

### Follow-up

Patients were scheduled to return for medical evaluation at 1, 3, 6, and 12 months after the designated intervention, at which time a 12-lead ECG was performed. Physical examination and patient reports of recurrence of symptoms were obtained at each visit. The FAQoL score, 24-hour Holter monitoring, and the MMSE were performed at baseline and 6 and 12 months.

### Study outcomes

The primary outcome was the absence of AF or atrial tachycardia, lasting at least 30 seconds, documented after the three-month blanking period. Pre-defined secondary outcomes were progression to persistent forms of AF, impact on quality of life, and complications. In case of recurrence of atrial arrhythmia documented by ECG or Holter monitoring, the date of the event was considered as the onset of symptoms. A major complication was defined as any adverse effect resulting in death, permanent injury, or requiring hospital treatment.

### Statistical analysis

The sample size was calculated to provide 80% power to show the superiority of ablation over drug treatment in a trial comparing the primary outcome, with an alpha level of 0.05 two-sided, assuming recurrence probabilities of 65% in the ablation and 30% in the drug therapy group. The planned sample size was 60 patients (30 in the AC group and 30 in the AA group). Qualitative characteristics were described according to groups using absolute and relative frequencies, and the association was verified using the Chi-square test, Fisher’s exact test, or likelihood ratio test. Quantitative variables were described according to groups using summary measurements (mean ± standard deviation or median and interquartile range) and compared between groups using the unpaired Student’s t-test or the Mann-Whitney U test according to the probability distribution assessed using the Kolmogorov-Smirnov test. The QVFA scores were described by groups throughout the assessment moments. The summary measures and the comparison between the groups and these moments were used in the analysis of variance with repeated measures, followed by Bonferroni multiple comparisons to verify between which moments the differences occurred. Drug-specific analyses in the study used generalized estimating equations with a binomial distribution and logit link function to compare groups during the assessment. Kaplan-Meier curves were created according to groups to estimate the time to recurrence of AF and compare the groups in the log-rank test. To perform the analysis, the IBM-SPSS software for Windows version 20.0 was used. P values <0.05 (two-tailed) were considered statistically significant.

## Results

Patients were randomized between September 2017 and March 2020 at a single tertiary cardiology center (InCor/Universidade de São Paulo, Brazil). Sixty patients, 30 men, and 30 women were included. Table 1 describes the characteristics of the population. There were no significant clinical differences between the groups, except for the higher burden of AF in the AC group (p <0.001). The average time from AF diagnosis to inclusion time was 4 years (Q1: 2; 3:8) in the entire population.

**Table 1:** – Baseline Characteristics of the Patients

Characteristics	PVAC N = 30	AAD N = 30	p
Age, years-old	71.1 ± 4.0	72.1 ± 5.1	0.40*
Male, n (%)	13 (43.3)	15(50)	0.60
Hypertension, n (%)	27 (90)	30 (100)	0.24
BMI, (Kg/m^2^)	28 ± 4.3	28.1 ± 5.5	0.21*
Diabetes mellitus, n (%)	5 (16.7)	10 (33.3)	0.136
Dyslipidemia, n (%)	12 (40)	20 (66.7)	0.038
Anticoagulants, n (%)	10 (33.3)	15 (50)	0.08&
Warfarin	3 (10)	7 (23.3)	
Dabigatran	10 (33.3)	6 (20)	
Rivaroxaban	3 (10)	0 (0)	
Edoxaban	4 (13.3)	2 (6.7)	
Prior TIA/CVA, n (%)	2 (6.7)	3(10)	0.639
Time since AF diagnosis, years (IQR)	5 (2; 10)	3 (2; 5)	0.12§
Time since last AF episode, months (IQR)	9.5 (1; 25)	6 (3; 25)	0.76§
AF burden in 12 months (IQR)	12 (6; 37.5)	3 (0.5;11)	<0.01§
Antiarrhythmic drugs, n (%)			0.26&
Propafenone	9 (30)	12 (40)	
Beta-blocker only	4 (13.3)	4 (13.3)	
Amiodarone	11 (36.7)	13 (43.3)	
Sotalol	4 (13.3)	1 (3.3)	
Prior AF cardioversion, n (%)			0.25&
Chemical	8 (26.7)	9 (30)	
Electrical	5 (16.7)	10 (33.3)	
Both	1 (3.3)	0 (0)	
CHA2DS2-VASc score, n (%)			0.43&
1	1 (3.3)	0 (0)	
2	9 (30)	9 (30)	
3	14 (46.7)	10 (33.3)	
4	5 (16.7)	8 (26.7)	
5	1 ( 3.3)	3 (10)	
LV ejection fraction, %	64 ± 5	62.8 ± 3	0.28*
LA size, mm	41 ± 4	40 ± 6	0.69*

AF: atrial fibrillation; BMI: Body mass index, TIA: transient ischemic attack; RSPV: right superior pulmonary vein; CVA: cerebral vascular accident; IQR: interquartile range; PVAC: pulmonary veins ablation catheter; AAD: right atrial appendix; LV: left vein; LA: left atrial. Chi-square test; & Likelihood ratio test; * Unpaired Student’s t-test; § Mann-Whitney’s test.

### Ablation Group

The 30 patients randomized to AC underwent ablation. Table 2 presents the characteristics of the procedure. In seven (23.1%) patients, ablation of the cavotricuspid isthmus was performed, in addition to isolation of the PVs. Nine patients (30%) were referred for repeat procedures due to recurrence of symptomatic AF. During the second ablation, PV reconnection was observed in all patients: eight involving all four veins and one involving three veins. No ectopic triggers outside of PV were detected using high doses of isoproterenol.

**Table 2:** – Characteristics of the Ablation Procedure

Characteristics	N = 15
Ablation duration time, m	155 ± 49
LA Time, m	74 ± 37
Fluoroscopy time, m	13 ±8
All 4 PV isolated, n (%)	21 (70)
**Number of PV radiofrequency applications, n (IQR)**	
LSPV	8.5 (6;10.25)
LIPV	4 (4;7)
RSPV	5 (4;8)
RIPV	5 (4;7)
Heparin IU per patients	12. 500 (10 000; 17 125)
Major complication, n (%)	1 (3.3)

*IQR: interquartile range; IU: international units; LA: left atrial; LIPV: left inferior pulmonary vein; LSPV: left superior pulmonary vein; PV: pulmonary vein; RIPV: right inferior pulmonary vein; RSPV: right superior pulmonary vein.*

### Antiarrhythmic treatment group

AA therapies throughout follow-up and their respective doses are described in Table 3 and Figure 1. No patient randomized to the AA group underwent AC.

**Table 3:** – Antiarrhythmic Drug Use in Both Groups at Different Moments

Drug	PVAC N = 30	AAD N = 30	p
**Propafenone, n (%)**	0.099	0.970	
Before randomization	9 (30)	12 (40)	0.417
After initial treatment	12 (40)	13 (43.3)	0.793
1 month	12 (40)	13 (43.3)	0.793
3 months	12 (40)	15 (50)	0.436
6 months	5 (16.7)	12 (40)	0.045
12 months	2 (6.7)	11 (36.7)	0.005
**Sotalol, n (%)**	0.690	0.962	
Before randomization	4 (13.3)	1 (3.3)	0.148
After initial treatment	4 (13.3)	0 (0)	0.017
1 month	4 (13.3)	1 (3.3)	0.148
3 months	4 (13.3)	1 (3.3)	0.148
6 months	2 (6.7)	1 (3.3)	0.550
12 months	1 (3.3)	1 (3.3)	1
**Amiodarone,**	<0.001	0.999	
Before randomization	11 (36.7)	13 (43.3)	0.598
After initial treatment	12 (40)	13 (43.3)	0.793
1 month	13 (43.3)	13 (43.3)	1
3 months	11 (36.7)	12 (40)	0.791
6 months	1 (3.3)	12 (41.4)	<0.001
12 months	3 (10)	12 (40)	0.007
**Any AAD, n (%)**	<0.001	0.952	
Before randomization	24 (80)	26 (86.7)	0.488
After initial treatment	26 (86.7)	26 (86.7)	1
1 month	27 (90)	27 (90)	1
3 months	26 (86.7)	27 (90)	0.688
6 months	8 (26.7)	25 (83.3)	<0.001
12 months	6 (20)	24 (80)	<0.001

*Generalized estimating equations with binomial distribution followed by Bonferroni multiple comparisons. P value comparison between moments of each drug. AAD: right atrial appendage; PVAC: catheter multipolar pulmonary vein ablation.*

**Figure 1:** Use of Antiarrhythmic Drugs in Both Groups at Different Moments of the Follow-up. PVAC: catheter multipolar pulmonary vein ablation.

### Primary outcome

In the AC group, nine (30%) patients underwent a second procedure: eight patients underwent the same technique (PVAC Gold) and one underwent the conventional PV isolation technique with an 8.0 mm catheter-directed by the Lasso catheter (J&J). After a second CA, two patients still had a recurrence of AF and were maintained on antiarrhythmic medication. At the end of a mean follow-up of 719 days (Q1: 566; Q3: 730), six (20%) patients in the AC group and 12 (40%) patients in the AA group (p = 0.119; Figure 3) had a recurrence of AF. Most patients in the AC group discontinued AAs during follow-up. At the end of the 12-month follow-up, 20% of the AC group had received AA (class IC or III) compared with 80% of the AA group (p < 0.001). In the AC group, there was a trend (p = 0.099) towards a reduction in the use of propafenone and a significant reduction in the use of amiodarone (p <0.001). Rates of AA use in the first 12 months of follow-up are represented in Table 3 and Figure 1.

**Figure 3:** – Meier Curve of Atrial Fibrillation Recurrence in the 2-Year Follow-up, p Value: Log-rank Test. PVAC: catheter multipolar pulmonary vein ablation.

### Secondary outcomes

One patient in the AC group and 5 patients in the AA group developed persistent AF (p=0.073) at the end of follow-up. Significant improvement in FAQOL scores was observed in both groups at the 6- and 12-month score assessments compared to their baseline assessments (Figure 3). Palpitations and shortness of breath scores improved at both assessments in the PVAC group, but only at 12 months in the medical treatment group. Patients without AF recurrence showed a trend towards improvement in the palpitation score (p = 0.052) and global score (p = 0.062) at 12-month follow-up. Hospital admission related to arrhythmia was recommended for electrical or pharmacological cardioversion in seven patients in the AC group and three patients in the AA group (p = 0.166). There was no change in the intellectual test score from the beginning of randomization compared to the 6 and 12, 10-month follow-ups. (Table).

### Complications

There was no significant difference between the two groups: three patients in the ablation group (10%), compared to six patients (20%) in the clinical group, p = 0.472. No patient undergoing CA experienced pericardial effusion during the procedure. One patient underwent electrical cardioversion due to unstable sustained ventricular tachycardia related to air embolism during transseptal access. One patient presented sinus node dysfunction and was treated with permanent pacemaker implantation. There were no vascular complications or phrenic nerve injuries immediately after the procedure. Eight patients (26.6%) who underwent PVAC Gold ablation presented radiological images compatible with CE. The patient with gas embolization also presented an image of cerebral ischemia on MRI, with complete reversal of symptoms within five days. One patient presented with transient loss of vision caused by occlusion of the left retinal vein one day after isolation of the pulmonary veins (arterial embolism excluded). Brain MRI was normal, and symptoms resolved within 30 days. Three patients developed esophageal ulcers, KCC 2B (9.9%), but no patients developed atrioesophageal fistulas. However, a 68-year-old patient presented with a non-healing ulcer in the second EDA, being hospitalized and treated with antibiotics and parenteral nutrition for 7 days, with complete resolution of the lesion.

During the average time of 12 months, six (10%) patients in the clinical group had to stop or change AA due to side effects. Four (6.6%) patients were using amiodarone and one using propafenone associated with a beta-blocker. The main side effects of discontinuing the antiarrhythmic drug were bradycardia (five patients - 16.6%) and hyperthyroidism (one patient - 3.3%).

## Discussion

To our knowledge, this is the first prospective, randomized study comparing the results of paroxysmal AF ablation with the use of antiarrhythmic medications in elderly patients. The main findings of this study were that isolation of the pulmonary veins with the PVAC Gold catheter was effective in maintaining sinus rhythm in most elderly patients; only one patient developed persistent AF and there was a favorable impact on quality of life. However, CA did not show statistical superiority when compared to treatment with antiarrhythmic medications. In general, there was good clinical tolerance among elderly patients to the procedures, but 30% required reintervention in the first 6 months after ablation, 20% of them required the introduction of medication to maintain the rhythm, three presented complications with clinical repercussions, and 25% presented signals of CE on MRI evaluation.

The benefits of maintaining sinus rhythm, with ablation or AA, not only for symptom relief, have been debated for a long time, particularly in older patients.^10^ The EAST-AFNET 4 study recently showed that the strategy of starting early Rhythm maintenance therapy (primarily with AA) in patients with AF was associated with a lower risk of death from cardiovascular causes, stroke, hospitalization for heart failure, or acute coronary syndrome relative to heart rate control during a follow-up period of more than 5 years.^11^ Consequently, current AF management guidelines recommend that CA be considered in patients with paroxysmal AF to better control symptoms and preserve this clinical condition in the long term.^1,2^

However, the beneficial effects of AF ablation in the elderly population still present controversial aspects.^3^ A pre-specified subgroup analysis of the CABANA study revealed lower AF recurrence rates with ablation than with drug therapy in the three age subgroups evaluated, with adjusted hazard ratio (aHR) of 0.47 (95% CI, 0.35–0.62) for patients younger than 65 years of 0.58 (95% CI, 0.48–0.70) for those between 65 and 74 years old and 0.49 (95% CI, 0.34–0.70) for those over 75 years old, suggesting that AF ablation is superior to antiarrhythmic treatment in preventing recurrence regardless of age.^12^ However, current guidelines suggest caution when considering ablation in elderly patients due to a lack of conclusive data.^1,2^

In our study, although the comparative curves of AF recurrence-free rates in the two treatment groups diverged throughout the follow-up (Figure 3), there was no statistical difference between patients treated by ablation (80%) concerning antiarrhythmic treatment. (65%, p = 0.119). This was due to the insufficient number of patients involved in the study, calculated based on the results of previous studies with younger patients that suggested a recurrence-free rate of 65% in the ablation group and 30% in patients undergoing clinical treatment.^13^

Although the recurrence-free rates of the ablation group presented the expected results, this was not the case for the clinical group, which presented results superior to those obtained by historical controls.^13^ A possible explanation for this finding is that class IA drugs, IC and sotalol were the main antiarrhythmics used in the studies as the basis for sample calculation, and amiodarone was avoided. On the other hand, in our study, 40% of patients were treated with amiodarone during follow-up. It is known that amiodarone is twice as efficient when compared to propafenone and sotalol, despite side effects and a considerable rate of drug interruption during long-term follow-up.^14^ Medication adherence has also been a limitation for clinical treatment in the real world. Thus, an additional influence on the efficacy of AA may be related to the “controlled trial effect”. Finally, patients in the AA group had a better clinical profile in terms of the number of AF recurrences before inclusion, despite randomization.

In relation to the quality of life assessed through the QVFA score, the CA group demonstrated a tendency towards improvement in the palpitation score (p = 0.052) and the global score (p = 0.062), the majority of which were without AAD. This is an important finding because maintaining sinus rhythm with ADD can represent a clinical problem for elderly patients, as in addition to AAD having the potential to develop adverse effects and drug interactions, these patients often have to use multiple medications to treat additional comorbidities, present significant sinus bradycardia, atrioventricular and intraventricular conduction disorders or ventricular dysfunction, which are common limitations for the use of AA in this population.^15^

Spontaneous asymptomatic CE has been implicated as a cause of dementia in patients with AF, and neurocognitive decline has been reported after AC for AF.^16^ However, even without documented neurological signs and symptoms, the clinical consequence of these lesions is still uncertain. However, failure to maintain sinus rhythm, regardless of the CA procedure, is also related to cognitive decline and even progression to vascular dementia.^17^ Post-ablation CE has been investigated using several techniques that present a wide variation in incidence, from 1.7% to almost 40%.^18^

One of the reasons we chose the PVAC Gold catheter in this study was the result of the PRECISION GOLD trial that used this catheter and demonstrated a low incidence (2.1%) of asymptomatic CE, without showing an impact on neurocognitive tests.^19^ However, another study published after we started our work by Keçe et al.^20^ documented a 23% incidence of asymptomatic CE in patients undergoing PV isolation with the PVAC Gold catheter compared to 6% in patients undergoing point-to-point ablation with the Thermocool catheter (p = 0.042). In this study, there was also no difference between the groups in relation to the neuropsychological tests conducted during follow-up.^20^

**Figure 2:** – Mean Value of QLAF Score, * means p Value < 0.05. PVAC: catheter multipolar pulmonary vein ablation.

The interpretation of asymptomatic CE risk becomes more complex when we analyze the Tokuda et al. study which compared the incidence of CE in 462 patients (mean age 59±10 years) undergoing CA for paroxysmal AF with different technologies: point-by-point RF (193), cryoballoon (168), RF Hot balloon (50) and laser balloon (51). Brain MRI was also performed one day after the procedure and showed asymptomatic CE in 30%, 24%, 34%, and 39% in the respective groups, with cryoablation showing a lower rate of embolization when compared to laser balloon ablation, but without statistical difference with RF techniques (p =0.14). In this study, multivariate analysis also showed a relationship between advancing age and increased occurrence of CE (p <0.001).^21^ These studies show that there is great variability in the occurrence of brain lesions after CA using the same catheter technology, suggesting that those findings may depend on how the technique is performed by the intervention group. In our series, one patient (3.3%) presented transient symptoms that resolved completely within 5 days, but the brain image persisted on the control MRI performed one week later.

Silent esophageal injuries after AC can progress to atrioesophageal fistula, a serious, rare, and feared complication. To avoid it, several strategies have been used and, although controversial, the most used method has been monitoring esophageal temperature and power adjustments during the application of RF to the posterior wall of the left atrium.^22^ Recent studies using these adjustments to minimize injuries Thermal esophageal ulcers show that they still occur in around 20% of the different CA techniques, and in the vast majority of cases, they are mild.^23,24^ In the present study, we detected asymptomatic 2B esophageal ulcers in three (9.9%) patients during systematic endoscopy. performed after the procedure. In one (3.3%) patient, the ulcer progressed at the second-week endoscopic evaluation, and the patient required hospital treatment with complete healing during endoscopic follow-up.

Our study also showed that three (9.9%) elderly patients had some important complication after AC with PVAC Gold: one (3.3%) patient had an acute stroke without long-term sequelae; another (3.3%) developed retinal venous thrombosis with complete recovery of vision; and another (3.3%) developed an esophageal ulcer that required hospital treatment for healing. These complication rates appear to be higher when compared to traditional RF ablation and cryoablation.^25^ Thirty percent of patients undergoing AC PVAC Gold required a repeat ablation procedure due to symptomatic recurrences of AF. As observed in other PVAC Gold studies, PV reconnection was present in all patients in whom the procedure was repeated.^26^ This is an important issue, as elderly patients are not willing to tolerate new hospitalizations and repeated procedures. Therefore, more effective strategies to promote durable PVI in the first procedure are desirable for these elderly patients.

### Clinical implications

Several randomized and controlled studies have shown that CA for AF is safe and superior to AA in maintaining sinus rhythm and preventing AF recurrence. Recent meta-analyses have demonstrated the superiority of AC over clinical treatment, including in the first treatment indication of patients with AF.^27,28^However, an evident advantage of CA has not been demonstrated in older patients.^3^ The higher incidence of heart disease, comorbidities, and the low inclusion rate of these patients in randomized clinical trials were considered possible reasons for these findings.^3,13^ Therefore, more specific investigations into the role of AC in this important subgroup of patients are still necessary, especially in this era of the introduction of new technologies that provide greater safety while maintaining effectiveness.^29^ In this sense, the data obtained in our study can be useful for further investigations in this area.

### Limitations

The study sample size was not sufficient to demonstrate the superiority of CA, a common observation in studies conducted on younger patients. Monitoring the recurrence of tachyarrhythmias with an implanted device could provide the burden of AF in different strategies, with an impact on disease progression and patients’ quality of life. The absence of a group under heart rate control limits the assessment of the benefit of improving quality of life in both groups. The MMSE score has limitations in assessing cognition, especially about executive function, and may not have captured more subtle cognitive differences Although previous studies showed similar results when comparing PVAC GOLD with point-to-point RF ablation, with the benefit of a faster procedure with PVAC GOLD,^4,5^ two recent randomized studies demonstrated a higher incidence of asymptomatic cerebral embolization and lower effectiveness of ablation with the PVAC Gold when compared to new generation point by point RF systems.^20,30^

## Conclusions

CA with PVAC Gold and the use of antiarrhythmic drugs promoted the maintenance of sinus rhythm in most elderly patients with symptomatic paroxysmal AF, with no statistical difference between them. Both groups showed improvement in quality-of-life scores, with no statistical difference in the rate of progression to persistent AF or complications. However, patients undergoing ablation with PVAC Gold showed a higher rate of asymptomatic cerebral embolization than expected. Additional studies using technologies with a better safety profile are needed to evaluate the benefits of CA in elderly patients with AF.
